# Diagnosis of alveolodental ankylosis in unerupted canines: one of the answers to why the canine does not come

**DOI:** 10.1590/2177-6709.25.6.019-025.oin

**Published:** 2020

**Authors:** Alberto Consolaro, Omar Hadaya, Mauricio de Almeida Cardoso

**Affiliations:** 1 Universidade de São Paulo, Faculdade de Odontologia de Bauru (Bauru/SP, Brazil).; 2 Universidade de São Paulo, Faculdade de Odontologia de Ribeirão Preto (Ribeirão Preto/SP, Brazil).; 3 Digital Center Radiologia (Maringá/PR, Brazil).; 4 Faculdade São Leopoldo Mandic, Departamento de Ortodontia (Campinas/SP, Brazil).

**Keywords:** Dental resorption, Alveolodental ankylosis, Replacement tooth resorption, Canines, Impacted teeth

## Abstract

**Introduction:**

Teeth frequently fail to erupt and situations arise that prevent the canines from reaching the occlusal plane.

**Objective:**

Discourse about the three situations in which the canine does not reach the occlusal plane, and remains unerupted; and at the same time, point how to make a safe diagnosis of alveolodental ankylosis - one of the three causes -, based on tomography.

**Conclusions:**

Ankylosis occurs in impacted teeth by atrophy of the periodontal ligament, including the epithelial rests of Malassez. The tomographic signs of alveolodental ankylosis in unerupted canines are the interruption of hypodense periodontal space, discontinuity of the lamina dura and its continuity with the root surface, which gradually loses its regular shape.

## RESUMO

**Introdução:**

Muitas vezes, a erupção falha, e ocorrem situações que impedem que os caninos cheguem até o plano oclusal.

**Objetivos:**

Discorrer sobre quais as três situações nas quais o canino não chega até o plano oclusal, permanecendo não irrompido e, ao mesmo tempo, destacar como se diagnosticar com segurança uma dessas três causas, a anquilose alveolodentária, a partir da tomografia.

**Conclusões:**

A anquilose em dentes não irrompidos ocorre pela atrofia do ligamento periodontal, incluindo os Restos Epiteliais de Malassez. Os sinais tomográficos de uma anquilose alveolodentária em caninos não irrompidos são a interrupção do espaço periodontal hipodenso, a descontinuidade da lâmina dura e a sua continuidade com a superfície radicular, que, gradativamente, perde sua regularidade.

## ORIGIN OF THE PERICORONAL FOLLICLE

**Figure 1:**
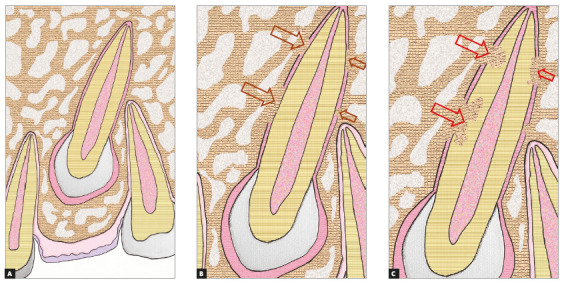
Schematic representation of alveolodental ankylosis in canine, in B, and of incipient replacement resorption, in C. In A, the pericoronal follicle and continuous periodontal space can be noticed.

The pericoronal follicle is adhered to the impacted canine (Fig 1). Previously, it was the organ that had already produced enamel and participated in the initial production of dentin, when it was named the enamel organ, which occurred during odontogenesis. 

In the most cervical portion of the crown that is already completely formed, the enamel organ gives rise to a structure that becomes independent. Because the shape of this structure is similar to that of the cuff or fold of a sleeve, it received the name of Hertwig's epithelial root sheath (HERS). It becomes dislodged, or detaches itself, from the pericoronal follicle to orient and participate in formation of the root and form the important epithelial network of the epithelial cell rests of Malassez, which are responsible for maintaining the periodontal space. 

After producing enamel and giving origin to Hertwig's epithelial root sheath, the mature enamel organ transforms its embryonic components into mature tissues, and as the set, receives the name of pericoronal follicle, and assumes another primordial function: that of being the main entity responsible for tooth eruption.

## IN WHAT WAY IS THE PERICORONAL FOLLICLE RESPONSIBLE FOR TOOTH ERUPTION?

Adhesion of the pericoronal follicle to enamel by the reduced epithelium of the enamel organ is performed by hemidesmosomes and assumes the following characteristics:

1. Without blood vessels, this epithelium is nourished by diffusion, from the tissue or interstitial liquid of the fibrous connective tissue that supports and surrounds it.
2. In this fibrous connective tissue organized in the form of capsules, there are numerous islets and remnant epithelial cords or vestiges of the dental lamina, which was eliminated by apoptosis soon after giving origin to the dental germ, but some of its cells persisted, and thus, organize themselves.
3. The reduced epithelium of the enamel organ plus the epithelial islets continually release the mediator EGF, or Epidermal or Epithelial Growth Factor.
4. EGF has the property of inducing bone resorption around it, and does not allow the bone to come near the enamel, that is, the tooth. In the root, EGF released by the Epithelial Rests of Malassez prevent the bone from arriving on the cement surface.

As odontogenesis occurs, by continually releasing EGF throughout the follicular tissue structure, the pericoronal follicle stimulates bone resorption around the crown, and thereby opens the pathway in bone for the tooth to make its way through to the occlusal plane. EGF interacts with other mediators, resulting in coordinated, efficient bone resorption.

The organ responsible for tooth eruption is the pericoronal follicle. If the pericoronal follicle is removed from any tooth, it loses its eruptive capacity, but if the root is removed, eruption continues normally. 

## WHY DO THE CANINES SOMETIMES NOT ARRIVE?

When the canines do not arrive, in general, the failure does not lie in the structure and function of the pericoronal follicle, but due to the following three situations that will be described: 1) Due to the lack of space for it and its crown; 2) Due to the inadequate position and direction of the long axis of the tooth; or 3) Due to the fact that alveolodental ankylosis has become established in the periodontal ligament.

## FIRST SITUATION

*There is no space for the canine and the pericoronal follicle together. If this space is provided, the canine comes through naturally, without applying traction.*

The position and direction of the canine frequently appear to be adequate based on the space available for its crown in the dental arch, but the tooth continues to be impacted. The crown does not erupt; this event is promoted by the pericoronal follicle and its mediators.

For canine eruption to occur, the mesiodistal distance of its crown plus the thickness of the pericoronal follicle on both sides need to be contemplated in terms of the space in the dental arch. In order to have a calculation parameter, it could be said that the space in the dental arch required for the canine to erupt normally must be equal to 1.5*x* the mesiodistal distance of the crown. 

This measurement is a point of reference that makes it possible to calculate what can be achieved in clinical practice; and does not represent a closed and unrestricted point, which could be smaller, but the closer this measurement were to being equal to the mesiodistal distance of the crown, the less chance there would be for this canine to arrive naturally in the dental arch.

If there were 1.5*x* the mesiodistal width of the canine in the arch, and the position were more or less favorable, the canine would come through alone, and not require the application of traction.

## SECOND SITUATION

*There is adequate space, and the canine does not arrive: traction is required.*

Providing adequate space, as mentioned in the previous item, if the canine does not come through naturally after two to three months, without applying traction, the cause must be related to the unfavorable position or the direction of the tooth. In this case the application of orthodontic traction would lead to arrival of the tooth in its place.

Orthodontic traction represents a movement equal to any other orthodontic movement - even more favorable, by being an extrusion controlled by adequate forces in favor of the periodontal fibers orientation. However, orthodontic traction must be preceded by the necessary, adequate and controlled surgical procedures, in order to prevent consequences due to:

1. An unfortunate surgical manipulation of the cementoenamel junction; consequently, there would be external cervical resorption induced by surgical exposure of the dentin window found naturally in all the teeth in this region. These windows are microscopic.
2. If the tooth to be submitted to traction were luxated without planning, in an unfortunate and inadequate manner, this would be transformed into a surgically induced dental trauma. Consequently - before or after the tooth arrives in the dental arch -, this would lead to ankylosis and replacement resorption, and consequent loss of the tooth.
3. Do not allow acids and the bracket bonding products to reach the cementoenamel junction, thereby exposing the dentin windows naturally present in all human teeth, including the canines.

When bonding brackets, the ideal would be to leave a “band” approximately 2-mm wide, with the pericoronal follicle intact on the cementoenamel junction.

## THIRD SITUATION

*There is inadequate space, the canine does not come through naturally, not even with orthodontic traction applied: in this case, planned and adequate surgical luxation is required.*

For luxation in canines, or other teeth, to have a good prognosis, this procedure needs to be most skillfully performed, in order to break only the points and areas of the alveolar bone adhered to the root. The movement must be firm and secure. Once luxation has been perceived, in order to check that it has been obtained, a delicate instrument is used for subtly evaluating whether the tooth has gained mobility. If this were not done, the procedure would be transformed into an intrasurgical dental trauma. 

Surgically-assisted luxation alone brings no benefits to patients; this must be followed by the application of normal orthodontic traction. Should this not be possible, the above-mentioned procedure must not be performed. During the process of orthodontic traction, the movement induced promotes remodeling and repair of the bony bridges and areas of ankylosis. This considerably increases the chance of ankylosis being reverted in areas of the ligament repopulated by Epithelial Rests of Malassez from the neighboring areas. 

In cases in which surgically-assisted luxation brought no benefit to the patients, even in teeth submitted to orthodontic traction, this needs to be taken into consideration at the time of planning the cases. These cases must be re-analyzed in an endeavor to discover where the failure occurred, which led to unsuccessful treatment.

When verification is required during the surgical procedure for performing intentional luxation of an ankylosed tooth, which will subsequently be submitted to traction, the tooth must not be moved in and out of the alveolus, an no exaggerated lateral movements of the luxated tooth must be promoted, because they would characterize dental trauma, and the consequences may be:

1. Persistence of the alveolodental ankylosis because of having killed more Epithelial Rests of Malassez, and evolution of the tooth to replacement resorption, of which the main cause is dental trauma.
2. External cervical resorption by intrasurgical dental trauma.
3. Calcific metamorphosis (CM) of the pulp due to partial lesion of the vessels that enter the dental pulp, during the maneuver.
4. Aseptic pulp necrosis, due to complete rupture of the vessels that nourish the dental pulp, characterizing a dental trauma.
5. Internal resorption, induced by dental trauma only.

## WHY DOES ANKYLOSIS OCCUR IN IMPACTED TEETH?

The bone cannot touch the tooth because, if this were to occur, it would include the tooth in the continuous process of bone remodeling, promoting resorption of the root and simultaneously forming bone in its place, characterizing replacement resorption (Fig 1). 

The entity that does not allow bone to naturally touch the tooth is the epithelial network, similar to a basket ball basket -with the tooth inside it- known as the Epithelial Rests of Malassez, which have the following physiological function, among others: maintaining the bone distant from the tooth by means of a periodontal space of between 0.2 and 0.4mm, with a mean of 0.25mm. Alveolodental ankylosis represents the union of bone, also the alveolar bone itself, with cement.^1^

In impacted teeth, the only cause is accidental dental trauma, interoperative or of any other nature, such as opening bottles with the teeth, bumping against the teeth with instruments, such as the laryngoscope and many other innocent procedures, such as personal contact during sport and leisure practices, or in fights and accidents. Dental trauma, when the tooth touches bone, promotes destruction of the Epithelial Rests of Malassez.

In impacted teeth, which remain in the bone environment for a longer time after their complete formation, since they remain unerupted and/or without space in the dental arch, excessive atrophy of the periodontal ligament may occur when there is a prolonged period of lack of use.

**Figure 2:**
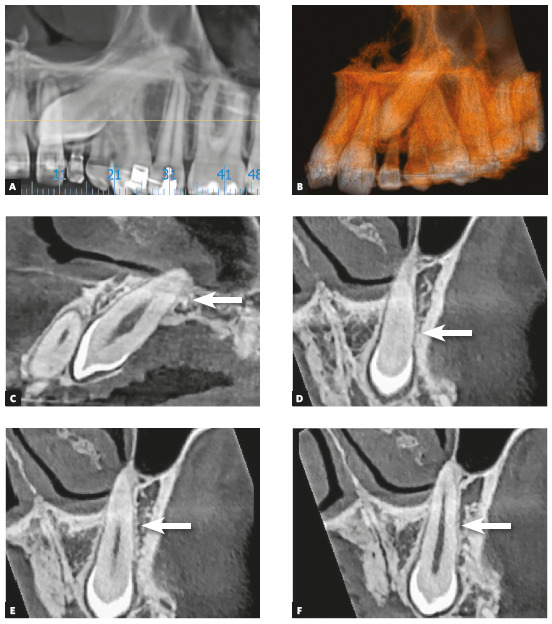
Alveolodental ankylosis in maxillary impacted left canine. First detectable tomographic signs are loss of continuity of periodontal space and lamina dura in points or segments, as in D to F, on distal surface (arrows), compared with periodontal space on palatal surface of root, in C (A = Panoramic reconstruction; B = 3D reconstruction; C = Sagittal reconstruction; D, E F = Coronal reconstructions).

**Figure 3:**
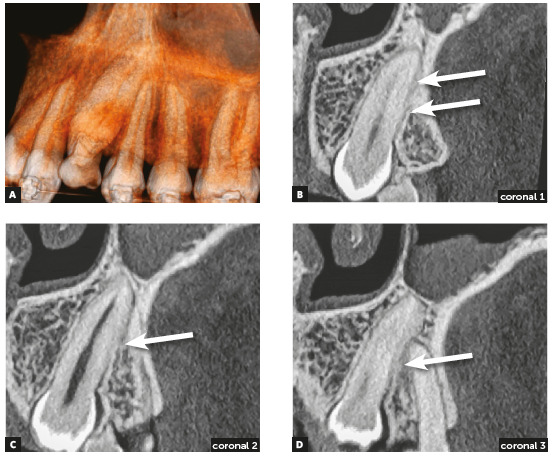
Alveolodental ankylosis in another maxillary impacted left canine. There is loss of continuity of periodontal space and lamina dura in segments on distal surface (arrows), compared with mesial surface of root. Superficial irregularities in root contour are outstanding, indicating incipient replacement resorption (A = 3D reconstruction; B, C and D = Coronal reconstructions).

**Figure 4:**
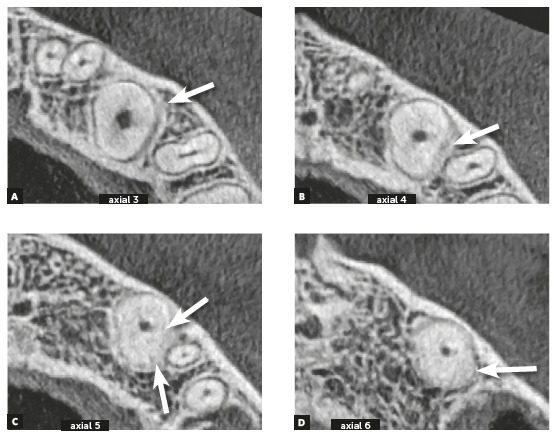
Alveolodental ankylosis in the same maxillary impacted left canine shown in Fig 2, now in axial reconstructions. There is loss of continuity of periodontal space and lamina dura in segments on distal surface (arrows). Superficial irregularities in root contour are outstanding, indicating incipient replacement resorption.

**Figure 5:**
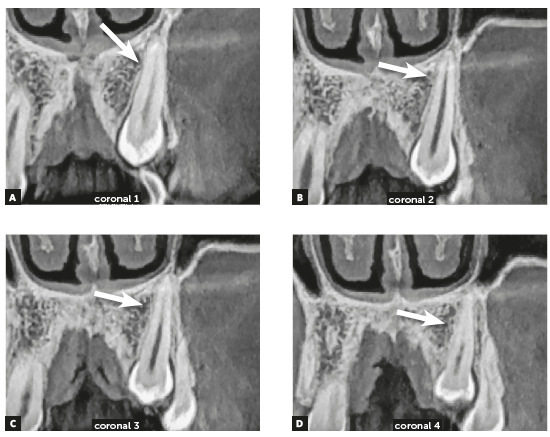
Alveolodental ankylosis in another maxillary impacted left canine, in coronal reconstructions. There is loss of continuity of periodontal space and lamina dura in segments on mesial surface (arrows). Superficial irregularities in root contour are outstanding, indicating incipient replacement resorption.

In teeth that remain unerupted for a longer period than the natural time, without function, the ligament becomes thinner than 0.2 mm, and the epithelial network without stimulus becomes even more delicate. Therefore, the bone that forms and resorbs all the time may trick the Epithelial Rests of Malassez into uniting themselves to the tooth. Initially this occurs at isolated points or isolated trabeculae, and gradually in larger areas, creating interfaces (Figs 1 to 5) of bone-tooth union.

Inevitably, alveolodental ankylosis develops gradually and naturally into replacement root resorption^1^. As from the point at which the bone came into contact with the root surface, their cells will consider the tooth as though it were bone, without distinction. While undergoing constant remodeling, the bone will resorb the tooth and slowly, progressively replace it, to the point where, some months and years later, there will be no more signs of a root at the site. This process is called replacement tooth resorption^1^ (Figs. 1 to 5). 

Alveolodental ankylosis and replacement tooth resorption are sequential phases in one and the same biological process, but we must not use the terms as synonyms. In replacement tooth resorption, loss of the tooth inevitably occurs in a slow and programmable manner. 

## TOMOGRAPHIC SIGNS OF ALVEOLODENTAL ANKYLOSIS IN IMPACTED CANINES

Due to variations in the position of unerupted teeth, with angulations and superimposition of images in radiographs, the safe diagnosis of alveolodental ankylosis and replacement resorption must be made on tomographic images. In tomographic images, the normal teeth exhibit a uniform and identifiable root surface. The lamina dura is shown as a radiopaque, hyperdense, well defined line, corresponding to the alveolar cortical bone. Between the tooth and lamina dura, a regular, uniform, well defined hypodense line is observed, which corresponds to the space of the periodontal ligament (Figs 1 to 5).

Seeking the continuous hypodense line of the periodontal space must be part of a meticulous analysis of the radicular and alveolar bone structures. The presence of lines and hypodense points in the periodontal space may correspond to bony trabeculae crossing it. The absence of periodontal space between the bone and root in small or larger segments in the tomographic sections indicates that there is alveolodental ankylosis. 

Some months or years in the situation of unerupted teeth may indicate the presence of atrophy due to lack of use of the periodontal ligament, with narrowing of the periodontal space, with increasing risk of alveolodental ankylosis and subsequent replacement resorption. The continuous deposition of bone may touch on the cement and determine alveolodental ankylosis.

In the tomographic images, the section and planes (axial, sagittal and coronal) are varied and allow a multiplicity of angles of observation, providing assurance for a precise diagnosis (Figs 2 to 5). The diagnosis of ankylosis and replacement resorption leads to loss of the tooth and generates most important clinical decision-making. Whether or not these details are described in the imagiologic exam report, it is always convenient and necessary for the clinician to check the data that was described by the imagiologist, and when in doubt, promote an exchange of information and experience with this professional.

In teeth in which ankylosis has developed into replacement resorption, the root surface loses its continuity, with bridges, lines or radiolucent spaces, and loses its radiopaque homogeneity or root hyperdensity at the site (Figs 2 to 5). The regularity of the line of the lamina dura is interrupted and mixed with these radicular areas, now equally irregular, as a result of interlocking with bone tissue. 

All these imagiological aspects are subtle, but sufficient for making a safe diagnosis. Even if the process of replacement resorption were a little more advanced, it would be clearly perceived in the axial, sagittal and coronal segments with respect to the pulp space, which would help to confirm that the process began in the external part of the tooth.

## FINAL CONSIDERATIONS

Impacted teeth atrophy the structures due to lack of use, and the periodontal space diminishes to an extent beyond the range of 0.2 to 0.4 mm, considered normal. This reduction places the tooth at risk because the bone may penetrate into the diminished epithelial network of the Rests of Malassez, originating the alveolodental ankylosis, followed by replacement resorption. Ankylosis represents one of the three causes of a canine failing to erupt in its place in the dental arch. Diagnosing alveolodental ankylosis in radiographs is extremely difficult, due to the distortions and superimpositions they present, but in tomography, the images are presented in various sections and planes (axial, sagittal and coronal). Therefore, they allow multiplicities of angles of observation, providing assurance for making a precise diagnosis. The precise and assured diagnosis of alveolodental ankylosis, and the most incipient form of replacement resorption resulting from it, requires the use of tomographic images.
